# Secondary Use of Health Data in Canada and the European Union: Expectations, Frictions and the Politics of Scale

**DOI:** 10.1111/1467-9566.70270

**Published:** 2026-09-29

**Authors:** Joseph Donia, Luca Marelli

**Affiliations:** ^1^ Department of Medical Biotechnology & Translational Medicine University of Milan Milan Italy; ^2^ Institute for Technoscience and Society York University Toronto Ontario Canada; ^3^ POLHIS – Research Center for the Ethics and Politics of Health Data Ecosystems University of Milan Milan Italy

**Keywords:** Canada, data spaces, European Health Data Space, European Union, governance, health data, secondary use

## Abstract

In this article, we trace expectations associated with efforts to promote secondary use of health data in Canada and the European Union. In Canada, we focus on initiatives enabling cross‐jurisdictional data access in a highly devolved federal system with parallel commitments to Indigenous data sovereignty. In the EU, we focus on the European Health Data Space, a new regulatory regime intended to facilitate access to health data for clinical care, research, policymaking and innovation across 27 member states. Drawing on a comparative analysis of policy documents and semi‐structured interviews across nine EU member states and four Canadian provinces and one territory, we find that despite institutional differences, secondary use in both jurisdictions is characterised by strikingly similar promissory vocabularies. We argue that these expectations are fundamentally ones of scale: They seek to render health data interoperable, comparable and usable to different ends. At the same time, we document shared frictions: between sovereignty and integration; between access and control; and between private assets and public goods. We understand these as spaces where competing valuations of health data are negotiated. We suggest that secondary use logics are progressively reorienting health systems, with implications for public value, equity and political collectives.

## 1 Introduction

Health systems today generate large volumes of digital data from an increasing variety of sources, including electronic medical records, personal devices and administrative processes. As in other sectors, efforts to promote access to and use of those data have risen steadily, shaped by policy imaginaries that strive towards more personalised, effective, efficient or timely approaches to care (Henwood and Marent 2019; Hoeyer 2023). The pursuit of different forms of value through health data integration has become more systematic over the last decade and increasingly features in national and regional health and innovation strategies (Gross and Geiger 2026; Reutter and Åm 2024). Internationally, governments and other actors have sought to make better use of data through new platforms, spaces and networks. At the supranational level, legislative efforts oriented towards realising the potential benefits associated with the access and use of digital health data have centred on the recently adopted Regulation on the European Health Data Space (EHDS), one of the most comprehensive and consequential regulatory initiatives targeting health data in the world (Marelli et al. 2023).

Perspectives from Science and Technology Studies (STS), including critical innovation studies (S. M. Pfotenhauer et al. 2019) and the infrastructuring of statecraft (Marelli et al. 2025), illuminate how health data sharing is frequently framed as a ‘cure’ for broader societal challenges such as health system sustainability, economic growth, industrial competitiveness, digital sovereignty and positioning within global AI economies. In this article, we extend these perspectives to demonstrate how expectations associated with secondary use of health data are scaled and stabilised and where they encounter friction in practice. We do so through a focus on two jurisdictions: Canada and the European Union (EU). We find that efforts to promote secondary use of health data in these jurisdictions are shaped by remarkably similar promissory vocabularies (e.g., innovation, better care, sovereignty, public value), yet produce very different institutional forms, tensions, contradictions and contestations.

We approach these tensions through the notion of friction (Bates et al. 2016; Edwards et al. 2011; Tsing 2012), which underscores that expectations and practices of health data access and use are never totalising. Rather than treating frictions as obstacles to overcome, however, we understand them as productive sites where competing values, logics and scales of governance are negotiated in practice. Friction allows us to examine how narratives are reworked as they encounter actual health systems and legal frameworks, while politicising otherwise invisible infrastructures and priorities (Donia et al. 2026; Star 1999).

To this end, this article makes three interrelated contributions. First, it offers one of the first comparative analyses of contemporary health data governance in Canada and the EU focused specifically on emerging health data spaces and platforms, extending a tradition of studying health systems as sites of contested value production. Second, by bringing together sociology of expectations, scale and friction, we demonstrate not only *what* is expected of health data but also *how* those expectations are produced, stabilised and reworked in practice. Third, we contribute empirically to debates about the political economy of health data integration by documenting how secondary use is becoming an increasingly hegemonic logic for health systems, particularly as interest in health‐related AI rises.

## 2 Secondary Use of Health Data in Canada and the EU

In health policy, secondary use generally refers to the reuse of health data for purposes beyond those for which they were originally collected (Safran et al. 2007). Depending on the jurisdiction and governance framework in question, these purposes may include scientific research, health system planning, public health surveillance, policymaking, quality improvement and commercial innovation activities. This broad understanding is reflected in contemporary governance frameworks, which increasingly treat secondary use as a jurisdictionally variable and contested category rather than a fixed technical one (OECD 2025); in the EU, for instance, the EHDS Regulation now defines secondary use in statute and enumerates the purposes it permits (European Commission 2025). In the following sections, we describe in greater detail the assemblage of rules, actors and infrastructures oriented to secondary use in Canada and the EU.

### 2.1 Canada

Health data in Canada are mostly subject to provincial privacy laws, including laws focused exclusively on health information. Where no such provincial laws exist, federal privacy law sets out requirements for collecting, protecting and using health‐related information. Definitions of health information vary by jurisdiction but converge around a source‐based understanding, which hinges on the setting in which the data were collected (e.g., hospital, clinic) and how they were obtained (e.g., routine clinical care). As a result, most secondary use in Canada is structured less as an individual rights‐based opt‐in/opt‐out regime and more as a collection of institution‐specific governance models that attempt to balance privacy protection with collective objectives related to health system design, scholarly research and policymaking. A typology of holders of routinely collected health and administrative data in Canada is reported in Supporting Information S1: Table S1.

Canada's strengths lie in its administrative datasets, which are linked intra‐provincially by encrypted personal identifiers via public health insurance numbers, encompassing nearly the entire population (∼41M). Canada's diversity has also been cited as a comparative advantage, with the highest proportion of first‐generation citizens and residents in the G7. Yet there remains immense variation in data access regimes, where researchers must navigate provincial, territorial and national privacy laws, divergent application requirements, fee systems, data processing environments and regional differences in resources and practices of data use. This became a flashpoint during the COVID‐19 pandemic, resulting in a renewed effort to collaboratively advance health data integration through the endorsement of a Pan‐Canadian Health Data Charter, dedicated funds from the federal government in exchange for provincial commitments to advancing data sharing, new legislation aimed at promoting health information interoperability (Bill S‐5) and the announcement of federal funding for the creation of a new ‘health sector data space’ (ISED 2026).

This has also been complemented by what has been described as a ‘stewardship’ turn in health data governance (Knoppers and Bernier 2026), which emphasises the management of health data as a resource to be mobilised through principles of responsible access and public‐interest considerations. This framing is often positioned against the dominant ‘custodial’ regime in Canada, where specific named roles and institutions control access to health information in accordance with regulatory obligations. Although both approaches may be oriented towards public benefit, in practice, they operationalise this aim through very different institutional logics and practices.

### 2.2 European Union

Various individual EU member states have sought to leverage health and social data for research and policymaking purposes. These include, most notably, the Nordic countries, which pioneered the establishment of population‐based health data registries in the 1960s and 1970s. More recently, member states have created their own health data ‘ecosystems’, ‘platforms’ and ‘hubs’ (see Supporting Information S1: Table S2). Like Canada, varying levels of digitalisation within and across countries and regions mean that some member states have more advanced infrastructures and population coverage than others. European health systems are also characterised by significant diversity in funding, organisation and delivery, underpinned by different social welfare models (Mossialos 2010).

Despite these differences (and in some ways because of them), the European Commission has made greater inroads into health. The organisation and delivery of healthcare remain national responsibilities, whereas the Union holds a Treaty‐based mandate to support, coordinate and supplement member states' actions in the field of public health. Since the 2010s, it has increasingly relied on this coordinating competence to structure cooperation, promote convergence and shape the conditions under which national health systems operate.

In addition, as commentators have noted, healthcare does not exist in a vacuum, and other EU‐level regulations and directives have shaped member states' health systems in important ways (Mossialos 2010). In particular, the promotion of the digital single market, coupled with the drive to digitalise health systems, has enabled the EU to influence national health systems in indirect but significant ways (Marelli et al. 2025).

Among the most ambitious EU‐wide initiatives explicitly oriented to digital health data is the EHDS, a new EU regulation setting out a comprehensive framework for primary and secondary use of health data across all EU member states, with the dual aim of strengthening patients' rights and access to their data while enabling large‐scale data access and use for research, innovation and policymaking purposes (European Commission 2025). The EHDS finds its origins in the European Strategy for Data, which articulates a broader ambition to establish a single market for data through the creation of common European data spaces across strategic sectors including agriculture, finance and energy.

## 3 Theoretical Approach

In this article, we bring together three related strands of scholarship to examine the politics of scale in secondary use of health data in Canada and the EU. First, we engage with sociology of expectations, and more specifically the performativity of expectations (Borup et al. 2006), to trace how assumptions embedded in policy documents, discussion papers, media reports and actors' statements come to shape action in relation to secondary use of health data. Although literature on expectations and closely allied terms (e.g., imaginaries, promises and hopes) is conceptually layered and diverse, all have to some extent been concerned with how unrealised futures come to bear on the present (Bazzani 2023; Geiger and Gross 2017; Henwood and Marent 2019; Petersen et al. 2014) and how those futures are often built on *past* expectations (Brown and Michael 2003). Wainwright et al. (2008), for example, read expectations through Bourdieu (1990) to foreground the performative role of ‘expectational capital’ in stem cell research, where scientists demarcate persuasive futures in the present to accumulate scientific authority and legitimise certain futures benefiting the field. In the same vein, contributions have also addressed the political economy of expectations, where temporalities and affects such as hope and fear intersect with commercial practices in health and medicine (Novas 2006; Petersen et al. 2014; Wainwright et al. 2008). These insights have proven especially influential in scholarship on digital health, which has examined how promissory discourses legitimise digital technologies in psychiatry (Pickersgill 2019), participate in the construction of data economies (Reutter and Åm 2024; Vezyridis and Timmons 2021) and support the data‐driven transformation of hospitals and healthcare more broadly (Gardner 2023; Medina‐Perea et al. 2024).

Through a comparative analysis, we extend sociology of expectations as it has been taken up in sociology of health by tracing how the movement of health data shapes contemporary health systems. Our engagement with the performative tradition of expectations in particular leads us to focus not only on how expectations justify or legitimise data sharing but also how they actively organise institutional choices by brokering relationships between allies through shared visions and vocabularies (Birch 2017) and by aligning interests and mobilising resources in ways that privilege some futures over others. Expectations also change as they travel, from local to regional, regional to national and national to international. Rather than focusing only on which expectations animate visions of seamless interoperability or data‐driven health systems, we also attend to *how* expectations are produced, stabilised and reworked as they circulate and are taken up in local practices. In relation to secondary use of health data, this includes attending to the role of standards (Webster and Eriksson 2008), the reflexive work of actors implicated in building data policies and infrastructures (de la Cruz et al. 2025), affective atmospheres of fear, hope or uncertainty (Adams et al. 2009; Del Vecchio Good et al. 1990) and ‘high’ or ‘low’ expectations (Swallow 2025), or ‘good’ and ‘bad’ ones (Tutton 2011).

In this sense, expectations may well be performative, but they do not always play out in ways that were initially envisioned or intended, and they often produce outcomes that diverge from, or even contradict, their original premises as they are scaled. Second, by scale, we refer to the different orders at which ideas and initiatives are expected to take hold. Scalable ideas or practices are those expected to travel without changing, involving a great deal of work and, often, the erasure of idiosyncrasies (Tsing 2012). This applies to material artefacts but also to expectations themselves. In policy, for example, S. Pfotenhauer et al. (2022) identify scale as a normative logic underpinning ‘grand challenges’, ‘mission‐oriented innovation’ and ‘living labs’. National strategies, regulations and digital health charters can also be understood in a similar way, dictating how problems should be solved and the expected benefits of doing so. At the same time, not all scaling entails flattening through standardisation (Tsing 2024); scale can also be mobilised in pursuit of more just or pluralistic futures (Lehtiniemi and Ruckenstein 2019).

Third, and related, efforts to scale digital health initiatives and the expectations associated with them often encounter friction (Ruckenstein and Lehtiniemi 2025; Tsing 2024). These frictions can occur in data formatting (e.g., meeting interoperability standards), regulations (e.g., interpretation, application), normative goals (e.g., trust) and scientific practices (e.g., disciplines or traditions) (Bates 2018; Laurent 2025; Van Rossem and Pelizza 2025). Åm et al. (2025), for example, focus on the ‘one‐stop‐shop’ vision for health data in Norway, Denmark and Finland, tracing the persistent ‘stickiness’ of fragmentation despite significant policy efforts and resources directed towards seamlessness in these settings. Similarly, Aula (2019) finds that expectations associated with digital health data are characterised by stubborn tensions between ideals of data‐driven health systems and the practical realities of healthcare infrastructures. Frictions, like expectations, are productive, and they are often used to justify political and policy reforms; without frictions, for example, there would be no calls for interoperability. This makes them essential to some kinds of work and a problem to be avoided in others. Frictions can therefore be used as a methodological device to open up debate and foreground the material, institutional and reflexive work required to make data ‘move’ to certain places or do certain things.

In this article, we focus on frictions encountered when expectations of health data sharing are scaled in Canada and the European Union. In particular, we set out to address the following questions:

- What expectations are associated with policy efforts promoting secondary use of health data in Canada and the European Union?
- How are these expectations reconfigured as they are scaled across different sites? What frictions do they encounter?
- What do these frictions reveal about how authority, value and political collectives are negotiated across sites of governance?

## 4 Methods

Comparative studies of Canada and the EU have been the focus of a body of scholarship spanning several decades. However, each jurisdiction is characterised by very different political histories, institutional arrangements and legal frameworks. Canada is a settler‐colonial federal nation state, whereas the EU is an ensemble of nation states in the form of an intergovernmental and partially supranational organisation. In Canada, the constitutional role for the organisation and delivery of healthcare lies with the 10 provinces and 3 territories, with a limited role for the federal government through health system financing, medical device regulations and for Canadian veterans, federal inmates and some on‐reserve First Nations. In the EU, health is primarily a competence of member states, which are themselves internally heterogeneous, with differing institutional traditions and, in several cases, regionally devolved models of healthcare governance.

Despite these important differences, shared tensions related to secondary use of health data make them particularly interesting cases to compare. First, Canada and the EU experience degrees of legal fragmentation in proposing and implementing new policies and resulting debates about the value and legitimacy of different political and economic actors. For example, provincial health information statutes in Canada and the coexistence of EU regulations with diverse national implementations generate uneven conditions for access, use and governance of health data, prompting contestation over who should benefit from data and under what terms. Second, these dynamics are further reinforced by disparities in economic resources and digital maturity across provinces, member states and regions, which translate into uneven capacities to build, govern and benefit from health data infrastructures. Finally, both are also experiencing a resurgence in interest in economic and political sovereignty, with health emerging as a strategic site where debates are playing out. This has included efforts to assert control over health data infrastructures, limit dependence on foreign technology vendors and frame data as a national or regional asset tied to competitiveness and security (Floridi 2020; Geist et al. 2025).

To examine these tensions in practice, a two‐case research design was adopted within the style and conventions of a Science and Technology Studies approach, acknowledging the co‐constitutive nature of a phenomenon of interest (e.g., secondary use) and its ‘context’ (Law 2017). The study progressed over approximately 1.5 years, enabling us to track how expectations shifted over time as policies and strategies were refined and took shape. In the EU, for example, the EHDS moved from proposal to regulation, with implementation work currently ongoing and extending into 2036. In Canada, bilateral agreements were signed, legislation was introduced, and national attention shifted more drastically to sovereignty and integration, which we describe in our results.

Data collection over this period involved qualitative semi‐structured interviews, document review and attendance of online events. In total, we conducted 43 interviews with policymakers, advocacy groups, innovators, data holders and data users in four Canadian provinces and one territory (total = 24), as well as Brussels and nine EU member states (total = 19). Participants were purposively sampled (Coyne 1997), with the aim of maximising richness and relevance to the phenomenon of interest (expectations associated with secondary use) across roles (e.g., data holder, data user, community group and policymaker) and jurisdictions (e.g., supranational, national, provincial and regional). Potential participants were identified through policy and professional networks and publicly available information, supplemented by referrals from interview participants. Interviews were conducted remotely via video call by the authors, audio recorded with participants' consent, transcribed verbatim and were on average 60 min in length.

Reviewed documents included policy texts, proposals, discussion papers, government strategies, news media reporting and lobby group communications related to secondary use at provincial, territorial, national and supranational levels (Canada = 100; EU = 102). In Europe, we focused specifically on the development and implementation of the EHDS and its interactions with existing national health systems. In Canada, we focused on broader bilateral and multilateral efforts to promote multijurisdictional secondary use of health data. Documents were identified through purposive sampling (focusing on landmark policies or initiatives), snowballing (references to other key documents) and interview participant referrals. A list of documents quoted in the Results sections of this paper can be found in Supporting Information S2. In‐text quotations of documents appear as ‘(SMB Reference #)’.

Materials were analysed abductively (Timmermans and Tavory 2012), focusing first on a review of prior literature, followed by informal reading of empirical materials, generation of first‐order expectations of health data access and use found in policy documents and interviews, and eventually ‘frictions’ as they were described by those we interviewed. Abductive analysis involves iterative movement between empirical materials and theoretical resources. Rather than treating theory as either a fixed starting point (deduction) or allowing themes to emerge purely from the data (induction), analysis proceeds iteratively between prior theoretical understandings and empirical material, enabling empirical ‘surprises’ to prompt refinement of theoretical premises and revisitation of findings. Qualitative analysis software NVivo supported the analysis of both verbatim interview transcripts and documents.

## 5 Results: Expectations of Secondary Use in Canada and the EU

In the following sections, we compare and contrast expectations related to secondary use of health data in Canada and the EU. We find that many expectations are shared within and across jurisdictions, while others appear in conflict or are mobilised to very different ends.

### 5.1 Canada

A recurring premise in interviews, policy documents, discussion papers, media reports and commentaries focused on secondary use in Canada and its provinces and territories is the under‐realisation of the country's potential, despite being an international leader in health research (Hobson 2025). Promissory discourses refer to ‘unleashing’ the power of health data to achieve different goals, all of which demand foundational shifts in governance, despite disagreements about exactly how that can be achieved.

#### 5.1.1 Supporting Evidence‐Based Policy

The earliest efforts to leverage administrative health data in Canada were primarily oriented to promoting evidence‐based policy, with the assumption that access to more data would facilitate faster or more precise responses to public health issues. This logic continues today through desires to build capacity to ‘inform public health advice’ and support ‘evidence‐informed decisions’ at both provincial and national levels via common standards and harmonised governance (SMB 3, 5). These ambitions are not only advanced by policymakers; patient groups in 2022 called on Ontario's provincial government to work with federal, provincial and territorial colleagues to:

[meet] the collective needs of people in Canada, to have a consistent, common, transparent, pan‐Canadian data strategy with interoperable architecture so that accessible data follow people in Canada…. to make evidence‐based health decisions and develop effective policies to serve everyone.

(SMB 9)

These calls occur against the backdrop of both the SARS outbreak and COVID‐19 pandemic, which represented significant public health events for the country. Data gaps are seen as threats to governability itself, meaning policymakers do not know ‘where to target programs’ or ‘how to improve those programs’ (SMB 1). These concerns are carried forward in more recent controversies related to large‐scale changes to public health policy instituted by the United States (SMB 8). At the same time, the prospect of interprovincial comparison enabled by harmonised data is clearly marked by political sensitivity, with commentators noting that provinces guarded their autonomy and remained resistant to being compared, as one leading data governance scholar highlighted:

Quebec is not going to send their data to Ontario. Everybody will say you can send your data here, but I'm not sending data there. And it's partly I think, I say small ‘p’ political because provinces don't necessarily want to be compared to one to another.

The issue is rarely lack of data, but which conditions permit actionable insights to be generated for health systems, without compromising regional political interests and priorities.

#### 5.1.2 Promoting World‐Class Scientific Research

Despite these challenges, commentators consistently frame Canadian scientific research as a comparative strength, describing Canada as an ‘internationally‐recognised leader in health data sharing for research’ (SMB 6, 7). Interview participants, however, repeatedly emphasised that successful multijurisdictional studies tended to occur despite, rather than because of, Canada's highly fragmented health data ecosystem. Projects were described as requiring ‘brute force by research teams who are intrepid and decide to do a multi‐province project’:

What people spend a lot of time on is negotiating access to data, doing all the procedural reviews, all of the documentation. And then when you get [the data] you have to do the deep cleaning and data preparation, and then you have to actually do your analysis. It's daunting. This is why we do single‐province studies to our detriment.

Canada's perceived standing in the world featured prominently, articulated through references to the country's declining position among high‐income countries in measures of healthcare performance or spending and speed of COVID‐19 genome sequencing, with ‘many countries ahead of Canada’ in recognising that ‘investments in data are a crucial component of health sector transformation’ (SMB 2). Within this promissory framing, national integration is expected to mobilise regional resources towards a future where Canada can ‘outshine any other jurisdiction in the world’ (CIHI 2025), providing domestic researchers and firms with competitive advantages. Comparative rankings and performance metrics play a central role in these future‐oriented narratives, in which the failure to fully mobilise health data is seen as undermining both scientific standing and health system performance.

#### 5.1.3 Better Access to and Provision of Care

In contemporary health systems, scientific research is entangled with a variety of valuation practices (Gross and Geiger 2023), most notably the expectation that data‐driven research should translate into tangible improvements to health system performance. These anticipated benefits extend beyond direct clinical use, to treatment of rare diseases, advancement of health equity and access, and alleviation of care providers' administrative burdens. As one senior hospital executive suggests:

We always should increase our knowledge. But for the end‐user, what are they getting back? Are fewer people dying? Am I saving money? I think we have to have a bit of a lens around how much money are we putting in, how much money we expect to get out, or clinical benefit we expect to get, and is it worth the trade‐off?

Expectations of improved care frame secondary use as imperative, repeatedly justified in policy documents through the slogan ‘data saves lives’ (SMB 4). Within this framing, secondary use is not understood as a departure from primary use but rather as its extension through other means, where research is expected to generate near‐real‐time improvements in care. As one respondent explained, this shift entails recasting health data from a ‘dangerous substance’ into a ‘national treasure’, valued for its capacity to circulate and generate action. That value is articulated both in normative terms, such as meeting the quintuple aims of patient experience, population health, reduced costs, health equity and improving workplace conditions, and in more instrumental terms, including the potential to offset system costs through additional revenue streams, such as data licensing. However, the means of doing so are equally important. Although many policy actors focused on the potential of data to improve access to and provision of care, including for structurally marginalised groups, others noted that it was equally ‘necessary to ensure this data is used ethically and effectively, enabling communities to control how their data are collected, shared and utilised’ (SMB 10). Aspirations for better access and care, while seemingly universal, are inseparable from ongoing negotiations over who controls health data, how they circulate and under which conditions their promised benefits are realised.

#### 5.1.4 Advancing Political and Economic Interests

Although intertwined political‐economic dimensions of secondary use subtly appeared across other expectations, a much more explicit turn has taken shape in recent years. Policy documents and health system actors increasingly frame health data as a strategic ‘asset’ and ‘untapped resource’ through which Canada's future competitiveness, sovereignty and national values can be secured (SMB 8). Here, sovereignty claims related to data simultaneously concern strategic autonomy and industrial strategy, especially through participation in emerging data economies. For example, expectations of digital sovereignty and control are entangled with the practical reality that many of Canada's health information infrastructures are owned and managed by large United States‐based firms (Geist et al. 2025). In practical terms, there remains an awkward tension between the desire to update Canada's privacy laws to prevent transfer to other countries, facilitate greater access for researchers and patients and safeguard Canadian values against a ‘multi‐tier, profit‐driven’ digital economy (SMB 2), while, at the same time, avoiding frictions that new regulations might introduce. For example, one senior policymaker notes:

At a time when Canada is under these external pressures, in which there are already concerns about productivity and whatnot, there needs to be a very compelling reason to create additional compliance costs.

Simultaneously, Indigenous data sovereignty in Canada represents a parallel legal commitment, normative goal and component of reconciliation, where health data are viewed not only as an economic resource but also as a broader means of self‐determination (Hale 2024). Privacy laws in British Columbia, for example, prohibited the First Nations Health Authority from accessing essential COVID‐19 data during the pandemic (Govorchin et al. 2026), exacerbating its effects in some communities. First Nations in Canada have made significant advancements in asserting greater autonomy over health, education and social services data, including enhanced authority over who may access these data and the purposes for which they may be used (First Nations Information Governance Centre 2024). There are recognised challenges to these expectations, however, including decisions about who is permitted to use data and when, and asymmetries when it comes to data storage and processing, with ministry budgets far exceeding those of many First Nations, and some provinces being much more advanced in these commitments than others.

### 5.2 European Union

Expectations related to secondary use of health data in the European Union are articulated through a distinct configuration of logics compared to Canada. These partly stem from the constitutional responsibilities of the European Commission versus member states when it comes to health and how those divisions are articulated through the EU's mandate to promote a European digital single market. The EHDS has become one key site where these debates play out, centring around a desire to advance economic and political sovereignty, respond to public health events effectively and efficiently and improve the health and care of the population (European Commission 2025).

#### 5.2.1 Creating a Single Market for Health‐Related Data

Expectations surrounding secondary use of health data at the EU level are strongly shaped by economic and political ambitions tied to market integration. The EHDS text explicitly cites the General Data Protection Regulation (GDPR) and national derogations as a hindrance to research and innovation, with the EHDS framed as an opportunity to reduce fragmentation by harmonising permissions, procedures and safeguards for accessing digital health data across borders (European Commission 2025). Harmonisation is expected to improve the functioning of the internal market by supporting interoperable EHR systems in conformity with the Union's legislation, while promoting a European data economy and home‐grown health innovation, particularly vis‐à‐vis the United States. As described by one data protection officer:

The opportunity to create a cohesive, free European space, independent from any other bloc and capable of conducting European research without necessarily following the U.S. model is, in my opinion, absolutely worthwhile… [But] you need to overcome all those localisms, resistances, and mistrusts.

At the same time, this expectation is accompanied by concerns that the EHDS could further entrench US‐based large multinational technology firms who have the resources to capitalise on cross‐border data access, especially as investment in AI intensifies. As contended by one prominent Member of the European Parliament involved in EHDS negotiations:

Technology companies and the pharmaceutical industry will have access to both anonymized and personally identifiable (only pseudonymized) patient files throughout Europe without the patient’s consent inter alia for product development, innovation and training artificial intelligence… [All these aspects] will expose European health systems to Big Tech.

#### 5.2.2 Promoting Scientific Research as a Driver of Innovation

Communications from the European Commission emphasise that the new data space will strengthen the European research ecosystem by providing researchers with access to larger volumes of health data, accessed more efficiently and at lower cost through designated national data access bodies rather than directly through institutional contact points (European Commission 2025). These expectations are particularly pronounced in areas where data scarcity has limited scientific research. Rare diseases are frequently invoked as paradigmatic cases where cross‐border access and use are necessary (SMB 16). ‘Real world data’ (RWD), cancer research and pharmaceutical development occupy a similarly prominent position in EHDS narratives (SMB 16). However, at this stage, the EHDS remains a work‐in‐progress. Even though the regulation has been adopted, the Commission timeline does not anticipate full implementation until the 2030s. This open‐endedness generates uncertainty and fuels concerns about how the EHDS framework will ultimately shape research practices and data access conditions (Donia et al. 2026). Among practitioners, for instance, concerns about data quality arose, with pharmaceutical associations expressing concern that ‘data not collected specifically for scientific purposes does not meet the very specific standards that are required for medicinal products’. Health data policy experts also expressed doubts as to whether the EHDS would succeed in overcoming existing legal and infrastructural fragmentation, including in relation to contentious topics such as informed consent in research. At the same time, researchers may continue to rely on existing national or disease‐specific access routes, leading to doubts about whether the EHDS will function as a transformative ‘one‐stop‐shop’ or a new layer with additional bureaucratic hurdles, as described by a data protection officer in one EU member state:

If a proposal comes in and they are only interested in my data, the pharma company can just contact me directly. Instead of going through Europe, they might prefer to come to me… Do [they] want to do it in one month or in six months?

#### 5.2.3 Improving the Health of the Union

Improving health outcomes and access to care tends to be more pronounced in the primary use provisions of the EHDS Regulation (Chapter II); however, health is frequently invoked as an indirect effect of secondary use, both in relation to care and information sharing for public health surveillance and policymaking, where ‘sharing data from one or several individuals can help treat, cure, or save another’ (SMB 16). Although the EHDS originates in the European Strategy for Data, the announcement of the European Health Union strategic policy framework in the early years of the COVID‐19 pandemic accelerated interest in a proposal for a health‐specific data space (European Commission 2022). The EHDS is cited in the Health Union announcement as ‘a key element in the creation of a strong and resilient European Health Union’, with the COVID‐19 pandemic highlighting ‘the imperative of having timely access to quality electronic health data’ (SMB 11). Timely access is expected to contribute to more efficient public health surveillance and monitoring, more effective management of future pandemics and a reduction of costs to ‘help to save more lives’ (SMB 11). The entanglement of health with policymaking, research and innovation is also pronounced in other key actors' materials, including, for example, the Standing Committee of European Doctors (SMB 15), who expect the EHDS to contribute to ‘the enhancement of the provision of health care by stimulating the availability of health data for scientific research’, and the European Patients’ Forum (SMB 17), who suggested that secondary use could help ‘improve health systems' sustainability, increase the quality, safety and patient‐centredness of health care’. These optimistic expectations are tempered by concerns about uneven implementation, digitalisation gaps, administrative burden on clinicians and the need to respect national traditions (SMB 15). Concerns were also raised about differences across disease types and regional attitudes towards data sharing, with some patient groups more enthusiastic than others about data sharing, as one organisational representative noted:

We were surprised because the mental health organisations were quite happy to share the data and not to have an opt‐in. And rare diseases, of course, it's much more important for them to share as many data as possible. [But] it also depends on the country they come from – how secure they are about the governments seeing all of this data, sexual health data… It really depended on the topic.

## 6 Discussion: Frictions and the Material Politics of Scale

Initiatives in Canada and the EU, although marked by distinct policy mechanisms and political actors, converge around shared expectations of more seamless health data access and use within and across borders. Below, we describe three tensions that address our research questions: the expectations shaping secondary use; when and how expectations encounter friction and are reconfigured as they scale; and what those frictions reveal about how authority, value and political collectives are negotiated in practice.

### 6.1 Between Sovereignty and Integration

Expectations of secondary use in Canada and the EU converge around more seamless access, integrated markets and harmonised infrastructures. However, this does not map neatly onto national and supranational arrangements. Instead, efforts to promote secondary use unfold across multiple scales at once, with health data governance becoming a site where political authority, economic ambition and regional identities are negotiated. In Canada, sovereignty is pursued at provincial, national, international and Indigenous levels. Most visibly, provinces and territories assert constitutional authority over health vis‐à‐vis the federal government, shaping debates about how and whether integration should proceed. However, sovereignty is also invoked in relation to foreign infrastructures; data may be stored domestically, but United States‐based service providers potentially render Canadian health information subject to United States law (O’Doherty et al. 2016). Parallel to these debates, Indigenous data sovereignty frames data governance as a matter of self‐determination, rather than market integration or coordination, raising practical‐political questions about how ambitions for sovereignty translate in provincially administered systems with national components.

In the EU, this tension is similarly multiscalar but institutionally distinct. The European project is premised on building a single market, yet healthcare remains a member state competence. At the same time, integration in domains such as digital health is increasingly pursued and justified not only through market logics but also through a broader discourse on ‘European values’ and on the need to reaffirm the very *raison d’être* of the Union, namely, a political project able to solve problems that individual states cannot address alone. As recent political narratives suggest, digital infrastructures and data governance initiatives are mobilised as instruments through which Europe seeks to define its identity, project its normative power globally and legitimise its existence in times of recurring crisis. Integration in health data is framed not merely as functional coordination but as part of a wider effort to sustain and rearticulate ‘Project Europe’ itself (Marelli et al. 2025).

### 6.2 Between Access and Control

In both Canada and the EU, expectations of seamless data use are mediated by institutionalised regimes of privacy, accountability and risk management, yet their underlying logics differ in important ways. In Canada, access is structured through custodial privacy regimes historically shaped by analogue imaginaries of physical cabinets and discrete record holders. Although digital infrastructures now allow data to be collected and stored with relative ease, statutory law continues to operate through distributed custodianship, where hospitals, clinics and specifically named individual roles act as gatekeepers. Within this context, privacy operates as a legal requirement embedded in statute, but it is also an institutionalised compliance regime shaped by risk management logics, including concerns about liability, reputational risk and administrative burdens associated with noncompliance (Baron and Lemmens 2023; Power 1997). As a result, data governance and privacy professionals often interpret rules conservatively, despite de‐identified data being subject to fewer protections in most provincial health‐specific privacy laws and arguably insufficient by contemporary standards (Spithoff 2022). In a rare agreement, nearly all actors we interviewed in Canada agreed on a need to shift from a custodial model to a ‘stewardship’ model, reframing data as something to use responsibly in pursuit of public benefit. Yet high‐profile cases in which de‐identified data were legally sold to data brokers, although still compliant with privacy law, have triggered public backlash in Canada (IPC 2022). Expectations of stewardship therefore hinge on a delicate balance that involves convincing publics that expanded use, including potentially commercial use, genuinely serves collective benefit, without arriving at a clear sense of the benefits to be pursued.

In the EU, access and control are structured through a very different legal regime, most notably the GDPR and its underlying principles. As in Canada, commentators have suggested that the regime has fostered a culture of caution among some data controllers, where the prospect of substantial administrative fines and reputational damage encourages more restrictive interpretations of what the law permits, even where data sharing for research is in principle allowed (Comandè and Schneider 2022; Sirur et al. 2018). Unlike the custodial model found in most provinces, the European framework foregrounds data subject rights and regulatory harmonisation. In this context, while the GDPR was intended to strike ‘the right balance’ between facilitating data flows and safeguarding individuals' rights (Marelli et al. 2020), the EHDS shifts the pendulum towards a more far‐reaching liberalisation of health data use. As such, the new data governance regime oscillates between expectations of individual patient control (in primary use) and increased use of data in research, innovation and policymaking activities at a broader EU scale (in secondary use), with unaddressed questions regarding how these tensions will be reconciled in implementation.

### 6.3 Between Private Asset and Public Good

Frictions between data as a private asset versus a public good most explicitly address what health data are meant to do and for whom. While it may be compelling to map these distinctions as a matter of public versus private *actors*, different conceptions of value coexist within institutions, organisations, policy frameworks and health systems more broadly. In Canada, this friction is particularly visible in debates over whether health data should be treated primarily as protected personal property, a collective public good or a strategic economic resource. Interviewees repeatedly described the need to balance public benefit with recognition that Canada possesses a ‘rich’ data ecosystem whose diversity holds considerable scientific and commercial value, especially in the face of strained and under‐resourced health systems. Health data are imagined simultaneously as essential to delivering better individual care and health equity and as a resource that can drive economic growth, with different ministries (e.g., industry vs. health), regulatory instruments and strategies materialising divergent understandings of what ‘valuable’ data consist of.

In the EU, the EHDS emerges from a more explicit economic justification, namely, the European Strategy for Data, which positions data spaces as engines of competitiveness and market integration. Here, conflicting notions of value arise between primary and secondary use but also among secondary users themselves: Researchers, pharmaceutical firms, digital health companies and public authorities in member states each mobilise distinct justifications for access. Invocations of protections under intellectual property law function as a mechanism to avoid obligations to share data through the EHDS, whereas new intermediaries such as Health Data Access Bodies are tasked with adjudicating these claims, even as their precise role, authority and compositions remain mostly unsettled (Quinn 2025). However, industry is not only a beneficiary but also a mandated contributor to the EHDS. Corporate actors holding health data may be required to share those data within the EHDS framework. Across both jurisdictions, the private asset/public good friction does not constitute a simple opposition but reflects competing performative claims about what health data are expected to do: safeguard rights, advance equity, stimulate markets or secure sovereignty. Taken together, these frictions reveal that authority, value and political collectives are not settled in advance of secondary use initiatives but are actively produced, contested and renegotiated through them.

## 7 Conclusion

In both Canada and the EU, health data initiatives are justified through universalist claims, yet these operate within highly differentiated social, institutional and normative landscapes. By focusing on frictions both unique to and shared by these jurisdictions, our analysis foregrounds two issues that we believe warrant further inquiry: (1) the political economy of health data integration and (2) secondary use as an increasingly hegemonic frame for the organisation of digitised health systems.

First, data integration initiatives are not merely policy reforms but emerging market‐making projects (Callon 2021). By standardising access procedures, establishing novel oversight and governance mechanisms and explicitly articulating fee policies, they actively reconfigure notions of value within regional, national and supranational health data ecosystems. Despite intending to promote fairness and competition, these emerging techno‐legal regimes risk privileging well‐resourced actors, reinforcing dependencies on private infrastructures and redistributing bargaining power away from local, regional or national institutions. Integration thus carries significant political‐economic implications. These dynamics are further intensified by the growing interest in artificial intelligence as a solution to long‐standing health system challenges, which depend on large‐scale, high‐quality health datasets. In this context, data integration also provides an infrastructural base layer for continued AI investment, potentially deepening existing asymmetries in access and control.

Second, and related, secondary use has become an organising logic for digital health systems more broadly. Initiatives oriented towards interoperability, linkage and reuse do more than blur distinctions between primary and secondary use; they progressively reorient health systems themselves towards secondary use goals, including but not limited to AI development. Health systems are sites of care delivery but also ‘platforms’ for anticipatory forms of value production that implicate an increasingly diverse set of actors. As expectations of future benefit become narrower, questions about whose purposes digitised health systems ultimately serve, and how competing values are negotiated in practice, become more urgent.

To this end, we suggest that future work explicitly embrace the multiple scales through which health data governance is pursued and realised. As health systems are repositioned as infrastructures for continuous data generation, circulation and reuse, future comparative and empirical research should examine how these transformations are translated, negotiated and resisted across institutional, regional and community settings, and how they reshape expectations surrounding care, participation and collective benefit. Attending to these dynamics can help move beyond abstract aspirations of seamless data circulation towards governance approaches that recognise integration as ongoing political, economic and organisational processes that must balance a number of competing goals and heterogeneous positions through which health data are assigned value in the first place.

## Author Contributions

**Joseph Donia:** conceptualization, investigation, writing – original draft, funding acquisition, methodology, writing – review and editing, formal analysis, project administration. **Luca Marelli:** conceptualization, investigation, funding acquisition, writing – original draft, methodology, writing – review and editing, formal analysis, project administration, supervision.

## Ethics Statement

This study received approval from the University of Milan Research Ethics Committee.

## Consent

All participants provided informed consent prior to taking part in this study.

## Conflicts of Interest

The authors declare no conflicts of interest.

## Permission to Reproduce Material

No material requiring permission has been reproduced from other sources.

## Supporting information


Supporting Information S1

Supporting Information S2

## Data Availability

The datasets generated during and/or analysed during the current study are available from the corresponding author on reasonable request.
