# Supplementary material for: Secondary Use of Health Data in Canada and the European Union: Expectations, Frictions and the Politics of Scale
Source: Sociol Health Illn. 2026 Sep 29;48(8):e70270. doi: 10.1111/1467-9566.70270 (PMC13624370; doi:10.1111/1467-9566.70270)
Supplement: Supplementary file 1 [file SHIL-48-0-s001.docx]

**Supplementary Material B**

**Primary Documents Cited**

The documents listed below formed a small part of the corpus analysed in this study. In-text citations in the main manuscript (e.g., SMB 1, 13) refer to the numbered entries below.

# Canada

## ***Government departments and agencies***

1. CIHI (2025) *Health Data Stewardship Framework*. Canadian Institute for Health Information. Available at: https://www.cihi.ca/en/about-cihi/corporate-strategies/health-data-stewardship/health-data-stewardship-framework (accessed 20 May 2026).

2. Public Health Agency of Canada (2021) *Expert Advisory Group Report 2: Building Canada’s Health Data Foundation*. November. Available at: https://www.canada.ca/en/public-health/corporate/mandate/about-agency/external-advisory-bodies/list/pan-canadian-health-data-strategy-reports-summaries/expert-advisory-group-report-02-building-canada-health-data-foundation.html.

3. Public Health Agency of Canada (2022) *Expert Advisory Group Report 3: Toward a World-class Health Data System*. May. Available at: https://www.canada.ca/en/public-health/corporate/mandate/about-agency/external-advisory-bodies/list/pan-canadian-health-data-strategy-reports-summaries/expert-advisory-group-report-03-toward-world-class-health-data-system.html

4. Health Canada (2026) The Government of Canada Introduces Legislation to Build a More Connected Health Care System. News release, 4 February. Available at: https://www.canada.ca/en/health-canada/news/2026/02/the-government-of-canada-introduces-legislation-to-build-a-more-connected-health-care-system.html

## ***Advisory, expert, and policy commentary***

5. Buckeridge D (2022) An evidence-informed vision for a public health data system in Canada. National Collaborating Centres for Public Health.

6. CCA (2023) *Connecting the Dots: Health Data Sharing in Canada*. Ottawa: Council of Canadian Academies. Available at: https://www.cca-reports.ca/reports/health-data-sharing-in-canada/ (accessed 9 February 2026).

7. CCA (2025) *The State of Science, Technology, and Innovation in Canada 2025*. Ottawa: Council of Canadian Academies. Available at: https://www.cca-reports.ca/reports/the-state-of-science-and-technology-and-innovation-in-canada/ (accessed 10 February 2026).

8. Waddell C (2025) Canada’s Code Red. Public Policy Forum. Available at: https://ppforum.ca/publications/canada-code-red/ (accessed 5 February 2026).

## ***Professional, industry, and civil-society organisations***

9. Canadian Patients’ Groups (2022) Patients Call for Pan-Canadian Health Data Strategy. 10 November. Available at: https://survivornet.ca/wp-content/uploads/2022/11/Patients-Call-for-pan-Canadian-Health-Data-Strategy-Premiers-November-10-2022.pdf.

10. Wellesley Institute (2025) *Learnings from Health System Partners Implementing Community Data Governance*. May. Available at: https://www.wellesleyinstitute.com/wp-content/uploads/2025/07/Wellesley-Institute-Report-with-CPAC_2025_Final.pdf.

# European Union and Supranational

## ***European Union institutions and legislation***

11. European Commission (2022) *European Health Union: A European Health Data Space for People and Science*. Brussels. Available at: https://ec.europa.eu/commission/presscorner/detail/en/ip_22_2711.

12. European Commission (2025a) European Health Data Space Regulation (EHDS) – Public Health. Available at: https://health.ec.europa.eu/ehealth-digital-health-and-care/european-health-data-space-regulation-ehds_en (accessed 11 February 2026).

13. European Commission (2025b) Frequently Asked Questions on the European Health Data Space – Public Health. Available at: https://health.ec.europa.eu/latest-updates/frequently-asked-questions-european-health-data-space-2025-03-05_en (accessed 10 February 2026).

14. European Parliament and Council of the European Union (2025) Regulation (EU) 2025/327 of 11 February 2025 on the European Health Data Space. Official Journal of the European Union, L series. Available at: https://eur-lex.europa.eu/eli/reg/2025/327/oj/eng (accessed 23 July 2026).

## ***Professional, industry, and patient organisations***

15. CPME (2022) *Standing Committee of European Doctors (CPME) Position on the European Health Data Space*. Available at: https://www.cpme.eu/api/documents/adopted/2022/11/cpme.2022-065.FINAL.CPME.position.EHDS.pdf.

16. EFPIA (2024) *EFPIA Position on the Regulation on the European Health Data Space (EHDS)*. Available at: https://www.efpia.eu/media/2t2dem05/efpia-position-on-ehds.pdf.

17. European Patients’ Forum (2022) *EPF’s Response to the European Commission’s Call for Feedback on the European Health Data Space*. Available at: https://www.eu-patient.eu/globalassets/news/20220720-ehds-call-for-feedback---final.pdf.
