# Supplementary material for: Secondary Use of Health Data in Canada and the European Union: Expectations, Frictions and the Politics of Scale
Source: Sociol Health Illn. 2026 Sep 29;48(8):e70270. doi: 10.1111/1467-9566.70270 (PMC13624370; doi:10.1111/1467-9566.70270)
Supplement: Supplementary file 2 [file SHIL-48-0-s002.docx]

**Supplementary Material A**

**Table 1: Health and Administrative Data Available for Secondary Use in Canada**

| **Infrastructure** | **Data Sources** | **Examples** | **Coverage** |
| --- | --- | --- | --- |
| EHR-derived research platforms | Participating hospitals and hospital networks | GEMINI (ON): participating hospitals | Patients treated in participating hospitals |
| Provincial administrative health data | Public health insurance plans; Ministries of Health | ICES (ON), PopData BC, MCHP (MB) | Near‑universal; residents eligible for provincial health insurance plan |
| Disease registries | Provincial cancer agencies; public health authorities; specialty clinical programs | Ontario Cancer Registry; BC Cancer Registry; CCDSS | Patients with specific diagnoses |
| Population cohorts & biobanks | Universities; research hospitals; CIHR-funded cohort infrastructures | Canadian Longitudinal Study on Aging (CLSA); CanPath; provincial cohorts (Ontario Health Study, BC Generations Project) | Volunteer participation |
| Cross‑sector linked health & social data | Various provincial ministries; regional health authorities; Statistics Canada | MCHP, PopData BC, ON & Statistics Canada | Varies by province |
| National institutes | Participating provinces and territories | CIHI | Users of publicly funded services across provinces and territories |
| National network of networks | Participating data holders | HDRN Canada | Residents of participating provinces and territories (federated, non-pooled) |

*** Acronyms**

CCDSS: Canadian Chronic Disease Surveillance System
ICES: formerly, Institute for Clinical Evaluative Sciences

MCHP: Manitoba Centre for Health Policy
GEMINI: originally, General Medicine Inpatient Initiative
CanPath: Canadian Partnership for Tomorrow's Health
CIHI: Canadian Institute for Health Information
HDRN: Health Data Research Network (Canada)

**Table 2: Select National & EU-Wide Health Data Spaces & Platforms**

| **Infrastructure** | **Description** | **Data Sources** |
| --- | --- | --- |
| French Health  Data Hub | National platform facilitating secure access to linked administrative and clinical health data. | National Health Data System (SNDS): health insurance claims, hospital discharge data, causes of death, disability data; linked clinical and administrative datasets |
| Findata | Statutory national data permit authority that centralizes authorization, coordination, and secure access to health and social data for secondary use. | Registers from health care providers, social services, population registers, disease registries, biobanks |
| Danish Health Data Authority | Central authority responsible for national health data governance, registry stewardship, and provision of secure access for research. | National health registries: hospital activity, prescriptions, primary care, civil registration system |
| Italian National Health Data Ecosystem | Federated national infrastructure integrating regional electronic health records and enabling interoperability for primary care, with emerging capacities for secondary use. | Regional electronic health records (FSE), prescriptions, discharge summaries, specialist reports, national health monitoring flows, interoperable metadata layer coordinated at the national level |
| European Health Data Space | EU-wide regulatory and infrastructural framework designed to enable cross-border primary and secondary use of health data. | Health data made available by Member States for secondary use via national health data access bodies |
